# Leucogen-induced pemphigus foliaceus: the first case report

**DOI:** 10.3389/fimmu.2026.1745063

**Published:** 2026-03-11

**Authors:** Ke Gui, Jingyao Liang, Aili Gao, Yuwu Luo, Xin Tian

**Affiliations:** 1 Department of Dermatology, Guangzhou Dermatology Hospital, Guangzhou, Guangdong, China; 2 Institute of Dermatology, Guangzhou Medical University, Guangzhou, China

**Keywords:** breastcancer, drug-induced pemphigus, HER2-targeted therapy, leucogen, thiazolidine

## Abstract

Drug-induced pemphigus (DIP) is a distinct subgroup of rare autoimmune blistering disorders, most commonly associated with thiol-containing medications such as penicillamine and captopril. Currently, an increasing number of pemphigus cases induced by non-thiol and non-phenolic drugs have been reported. Herein, we describe a 61-year-old woman with stage IV breast cancer who developed pemphigus foliaceus (PF) after receiving leucogen administration during anti-HER2 (THP) therapy. Cutaneous lesions resolved after leucogen withdrawal and corticosteroid treatment but recurred upon drug re-administration, confirming a probable causal relationship. Histopathological examination and direct immunofluorescence confirmed PF, characterized by intercellular IgG deposition and elevated anti-desmoglein 1 (anti-Dsg1) autoantibody titers. This case highlights that leucogen, a sulfur-containing agent with a thiazolidine-related structure, may represent a potential trigger for DIP, although the underlying mechanism remains speculative.

## 1 Introduction

Drug-induced pemphigus (DIP) represents a distinct subgroup of autoimmune blistering disorders induced by pharmaceutical agents. The most well-known causative agents include thiol compounds such as penicillamine, captopril, and bucillamine (1), which may disrupt keratinocyte adhesion via sulfhydryl-mediated impairment of desmosomal proteins (2). However, an increasing number of DIP cases have been linked to non-thiol and non-phenolic medications, which account for approximately 41.9% of reported cases, such as gliptin and liraglutide (3, 4). The pathogenic mechanism underlying these non-thiol, non-phenolic drug-induced cases remain incompletely understood. Herein, we present a case of 61-year-old woman who developed recurrent pemphigus foliaceus (PF) following combination therapy with paclitaxel, trastuzumab, pertuzumab (THP regimen), together with leucogen. Based on the temporal relationship between drug administration and lesion development, leucogen-induced PF was suspected. To our knowledge, following a systematic search of PubMed, Embase, and Web of Science databases, leucogen—a sulfur-containing leukopoietic agent—has not been previously implicated in the induction of pemphigus. Accordingly, this report documents the first case of PF associated with leucogen exposure. This clinical observation suggests a potential pathogenic link between its thiazolidine-related structure and the development of autoimmune cutaneous reactivity.

## 2 Case report

A 61-year-old woman with stage IV breast cancer presented to our hospital with a two-month history of erythematous, flaccid blisters accompanied by itching and pain on the neck, which developed following her third cycle of THP regimen administered every 3 weeks, combined with leucogen 20 mg three times daily. The patient had no prior history of autoimmune or allergic diseases.

**Figure 1:**
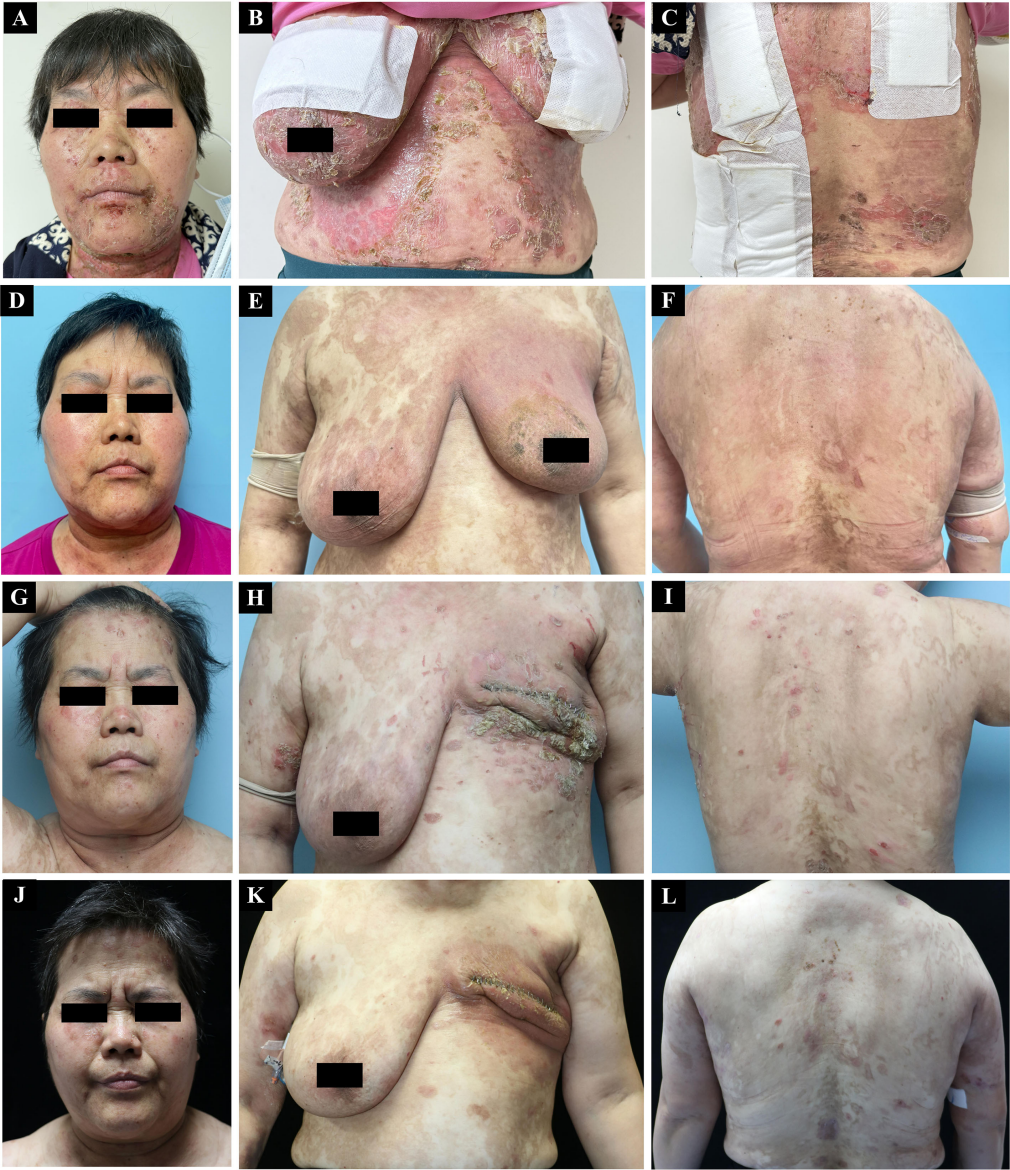
**(A–C)** Initial presentation showing flaccid blisters and erosions on neck and face. **(D–F)** Post-treatment resolution with residual pigmentation. **(G–I)** Recurrent lesions after leucogen re-exposure. **(J–L)** Resolution following drug discontinuation.

**Figure 2:**
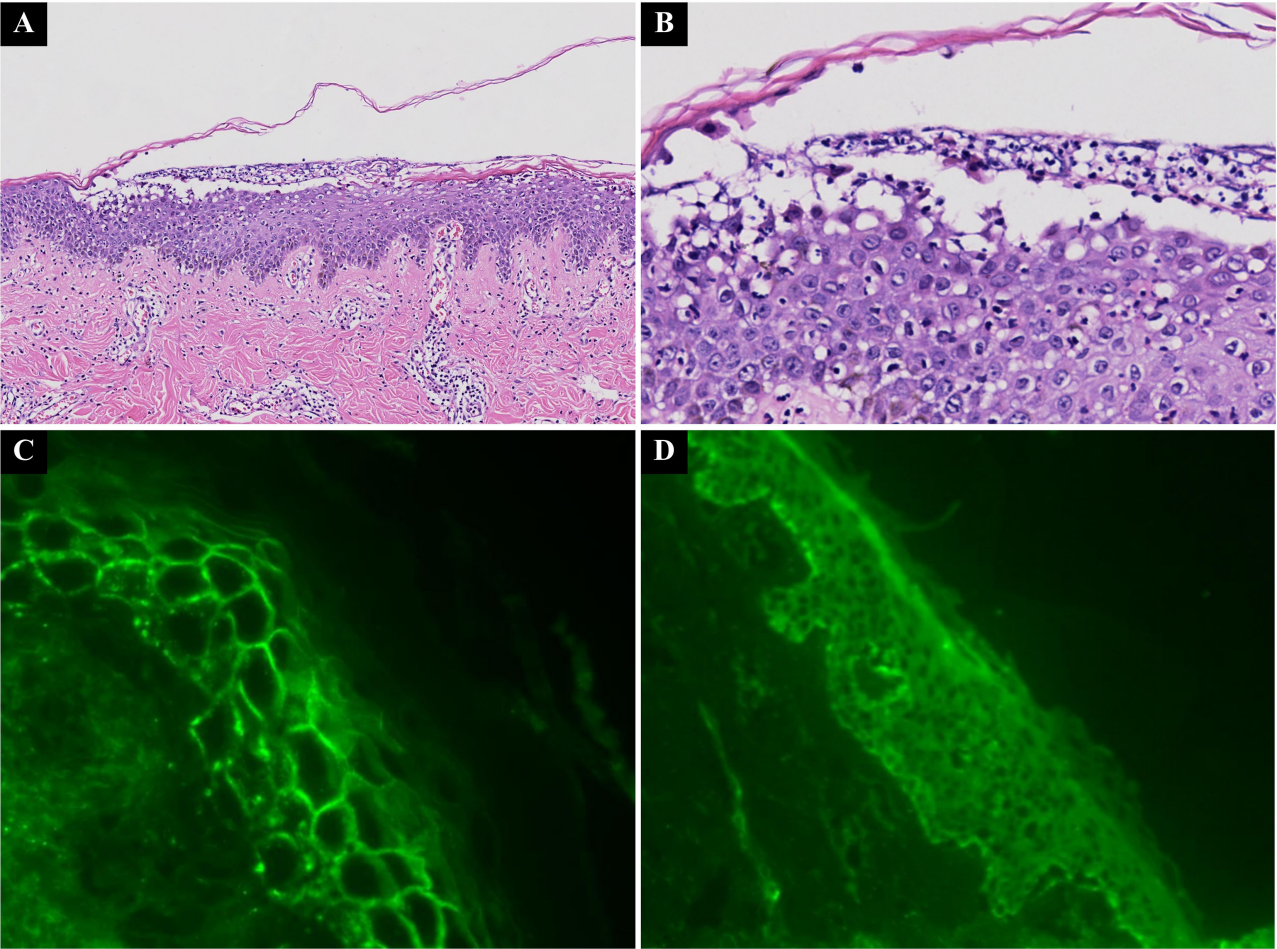
**(A, B)** Histopathology of a pustule biopsy showed subcorneal pustules with neutrophils and eosinophils, spongiosis, and lymphoplasmacytic infiltration in the superficial dermis (HE A×100; B×400). **(C, D)** Direct immunofluorescence shows intercellular IgG deposition and weak C3 positivity, while IgA and IgM are negative.

Physical examination revealed widespread erythema, erosions, and yellowish crusts involving the seborrheic areas of the face, neck, and upper back, with a positive Nikolsky sign (**Figures 1A–C**). To standardize disease severity assessment, the Autoimmune Bullous Skin Disorder Intensity Score (ABSIS) was calculated, yielding a score of 51. Laboratory testing showed leukocytosis (WBC 33.24 ×10^9^/L) and an elevated C-reactive protein (CRP) level (26.5 mg/L). Anti-desmoglein 1 (Dsg1) antibody levels were markedly elevated (> 6000 AU/mL; reference range: 0–20 AU/mL). Flow cytometric analysis of peripheral blood lymphocytes revealed normal NK cell counts (CD56^+^CD16^+^:8.11%) and elevated B cell counts (CD19^+^:21.85%). Bacterial culture of wound exudate identified *Staphylococcus aureus*. Tests for antinuclear antibodies (ANA), viral infections, and hepatitis B were negative. Histopathological examination of a pustule biopsy from the back showed subcorneal pustules containing neutrophils, eosinophils, subgranular vesicle, intercellular spongiosis among prickle cells, extensive lymphocytes and neutrophils migrating into the epidermis, and lymphoplasmacytic infiltrate around superficial dermal blood vessels. A small number of neutrophils and eosinophils were also observed in the surrounding tissues (**Figures 2A, B**). Direct immunofluorescence revealed intercellular IgG deposition in a chicken-wire pattern and weak C3 positivity (**Figures 2C, D**), confirming the diagnosis of PF.

All suspected medications, including leucogen, were discontinued. The patient was prescribed intravenous methylprednisolone 40 mg once daily, oral ciprofloxacin 10 mg once daily, and a single intramuscular injection of methotrexate 10 mg. Following initiation of this therapeutic regimen, the patient exhibited clinical improvement, with the ABSIS decreasing to 19.5. Notably, the ABSIS score remained relatively elevated at discharge due to extensive post-inflammatory hyperpigmentation, despite the absence of active blisters. The patient was then started on a tapering course of triamcinolone, beginning at 24 mg once daily, with the dose reduced by 8 mg every two weeks. At follow-up visits, the skin lesions completely disappeared and only post-inflammatory pigmentation remained (**Figures 1D–F**). Meanwhile, THP regimen was continued due to the necessity of maintaining breast cancer treatment during the corticosteroid taper. The patient did not experience any adverse side effects.

However, two months later, leucogen was reintroduced following breast tumor surgery. Within two weeks, the patient developed scattered erythema and flaccid blisters on the face again (**Figures 1G–I**). The ABSIS was 27, and anti-Dsg1 antibody levels remained significantly elevated (> 6000 AU/ml). Following discontinuation of leucogen and continuation of trastuzumab and pertuzumab therapy (HP regimen), the lesions gradually resolved with corticosteroid therapy, and the ABSIS decreased to 17.5, similar to the response observed during the initial episode (**Figures 1J–L**). Most recently, flow cytometric analysis of peripheral blood lymphocytes revealed an increase in NK cell counts (CD56^+^CD16^+^) from 8.11% to 34.74%, while B cell counts (CD19^+^) decreased to 3.74%. Based on the recurrence of PF upon leucogen rechallenge, a probable causal relationship between leucogen and PF was established.

## 3 Discussion

Idiopathic PF is generally considered to arise from a spontaneous breakdown of immune tolerance to Dsg1 in genetically susceptible individuals, in the absence of an identifiable exogenous trigger (5). This breakdown leads to a sustained but antigenically restricted autoimmune response targeting Dsg1. In contrast, DIP is thought to be initiated by exogenous pharmacologic factors that act as immunologic triggers rather than primary autoantigens. At least three groups of drugs are recognized as potential culprits in the development of DIP: thiols, phenols, and non-thiols/non-phenol. Thiol-containing drugs are thought to interfere with desmoglein function and activate proteolytic enzymes, whereas phenolic drugs may promote acantholysis via complement activation and protease modulation (2). For non-thiol/non-phenol drugs, the proposed mechanisms are more heterogeneous and may include antigen modification, hapten-like effects, or disruption of immune tolerance (1, 6). Leucogen, classified as a non-thiol/non-phenol drug, may therefore act as an immunologic trigger rather than a direct pathogenic effector. A thorough evaluation of the patient’s medication history is pivotal for diagnosis, with cessation of the offending agent serving as the mainstay of therapy. The patient experienced two episodes of pemphigus eruption, providing a clear clue that leucogen was the likely inducer.

Leucogen, a cysteine derivative, is widely used for the prevention and treatment of leukopenia and thrombocytopenia by stimulating bone marrow hematopoiesis and belongs to the non-thiol/non-phenol category (7). Common adverse events associated with leucogen include nausea, diarrhea, and neutropenia. Bone pain, myalgia, and arthralgia are more typically associated with granulocyte colony-stimulating factor therapy (7, 8). Although leucogen is not a classical thiol compound, its molecular structure contains a thiazolidine ring with a sulfur atom, which shares structural similarity to thiol-containing agents such as penicillamine. A literature review identified several drugs containing a thiazolidine ring or related structures, including penicillin, amoxicillin, ampicillin, linezolid, and piperacillin–tazobactam (6, 9). These agents have been reported to induce pemphigus, most commonly PF. Drawing on these structural and clinical parallels, we hypothesize that leucogen may contribute to the development of PF through indirect immunologic mechanisms, such as metabolic modification of epidermal antigens or hapten-like effects that facilitate autoantibody generation. While this hypothesis remains unvalidated experimentally, the clear temporal correlation between leucogen exposure and the two distinct PF episodes in this patient supports leucogen as the causal trigger.

Additionally, the potential contribution of the patient’s breast cancer history and prior THP regimen remains uncertain, raising questions about potential synergistic effects that could increase the risk of adverse reactions, including PF. Paclitaxel was excluded as a causative agent, as it was not administered during the second episode. The combination of pertuzumab and trastuzumab, both anti-human epidermal growth factor receptor 2 (HER2) monoclonal antibody, has been associated with adverse reactions such as neutropenia and diarrhea (10, 11). Regarding skin-related adverse reactions, trastuzumab etamsine (T-DM1) and trastuzumab deruxtecan (T-DXd) exhibit distinct characteristics: T-DM1 predominantly causes cutaneous toxicities such as spider angiomas and telangiectasia, while T-DXd is more frequently associated with hair loss (12). Beyond HER2 signaling inhibition, anti-HER2 monoclonal antibodies are known to activate innate immune responses through antibody-dependent cellular cytotoxicity, leading to natural killer (NK) cell activation and release of proinflammatory mediators (13). Emerging evidence supports a role for NK cells and their crosstalk with dendritic cells in the immunopathogenesis of pemphigus (14), and elevated circulating NK cell populations (CD56^+^CD16^+^) were observed in our patients. Importantly, however, continued trastuzumab and pertuzumab therapy in the absence of leucogen exposure did not result in pemphigus recurrence. This key clinical finding indicates that trastuzumab and pertuzumab as potential immunologic enhancers rather than primary triggers in this case.

Paraneoplastic pemphigus(PNP) was carefully considered in the differential diagnosis given the presence of an underlying malignancy but was ultimately excluded based on both clinical and immunopathological findings. Clinically, the patient lacked severe mucosal involvement, palmoplantar lesions, and systemic manifestations such as fever or bronchiolitis obliterans—hallmark features of PNP (15). Immunologically, anti-desmoglein 3 antibodies were negative, and direct immunofluorescence did not demonstrate the characteristic IgG and/or C3 “double-band” deposition along the basement membrane zone. Furthermore, no clear correlation was observed between cutaneous disease activity and tumor burden, as blistering and erosive skin lesions recurred despite tumor resection and ongoing chemotherapy and targeted therapy. The patient’s favorable response to corticosteroid therapy further supported a diagnosis of DIP rather than paraneoplastic disease.

A limitation of this report is the absence of post-treatment anti-Dsg1 autoantibody titers following complete clinical remission. The patient was lost to further serological follow-up after symptomatic recovery, precluding analysis of whether antibody levels normalized or persisted at low levels.

Leucogen may represent a novel non-thiol sulfur-containing trigger for drug-induced PF. Clinicians should remain vigilant when prescribing this agent, especially in patients who have malignancy and are receiving concurrent immune-modulating therapies. The triggering/compounding of pemphigus can have multiple causes, such as malignancies. Further studies are warranted to elucidate the immunopathogenic mechanisms of leucogen-induced PF and its cross-reactivity with thiol-based compounds.

## Data Availability

The original contributions presented in the study are included in the article/supplementary material. Further inquiries can be directed to the corresponding authors.
